# Structure Design of GFRP Composite Leaf Spring: An Experimental and Finite Element Analysis

**DOI:** 10.3390/polym13081193

**Published:** 2021-04-07

**Authors:** Linlin Ma, Jingwu He, Yizhuo Gu, Zuoguang Zhang, Zechuan Yu, Ao Zhou, Lik-ho Tam, Chao Wu

**Affiliations:** 1 School of Materials Science and Engineering, Beihang University, 37 Xueyuan Road, Beijing 100191, China; mllin6@126.com (L.M.); benniegu@buaa.edu.cn (Y.G.); zgzhang@buaa.edu.cn (Z.Z.); 2 School of Aeronautic Science and Engineering, Beihang University, 37 Xueyuan Road, Beijing 100191, China; hejingwu@buaa.edu.cn; 3 School of Civil Engineering and Architecture, Wuhan University of Technology, Wuhan 430070, China; zecyui@whut.edu.cn; 4 School of Civil and Environmental Engineering, Harbin Institute of Technology, Shenzhen 518055, China; zhouao@hit.edu.cn; 5 School of Transportation Science and Engineering, Beihang University, 37 Xueyuan Road, Beijing 100191, China

**Keywords:** composite leaf spring, structural design, mechanical properties, finite element analysis, filament winding process

## Abstract

Due to the high load-bearing capacity and light weight, composite leaf spring with variable width and variable thickness has been increasingly used in the automobile industry to replace the conventional steel leaf spring with a heavy weight. The optimum structural design of composite leaf spring is particularly favorable for the weight reduction. In this study, an effective algorithm is developed for structural optimization of composite leaf spring. The mechanical performance of composite leaf spring with designed dimensions is characterized using a combined experimental and computational approach. Specifically, the composite leaf spring with variable width and variable thickness was prepared using the filament winding process, and the three-dimensional finite element (FE) model of the designed composite leaf spring is developed. The experimental sample and FE model of composite leaf spring are tested under the three-point bending method. From experimental and simulation results, it is shown that the bending stiffness of the designed leaf spring meets the design requirement in the automotive industry, while the results of stress calculation along all directions meet the requirements of material strength requirement. The developed algorithm contributes to the design method for optimizing the stiffness and strength performance of the composite leaf spring.

## 1. Introduction

**Figure 1:**
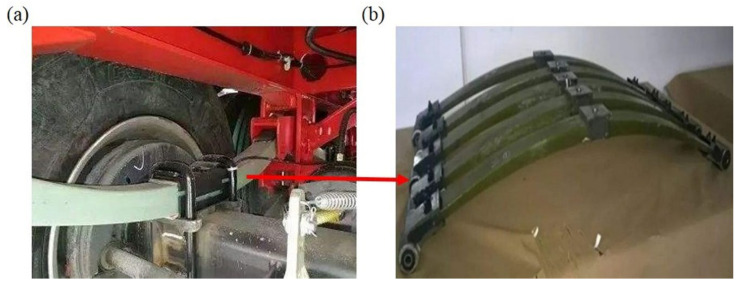
(**a**) Installation environment and (**b**) typical structure of composite leaf spring.

In the automobile industry, the quest for vehicles with energy conservation and pollution reduction is of vital importance due to the intensified market competition, global energy crisis, and the strict emission regulations required by environmental laws [1,2,3,4]. The automobile emission is the major contributor to environmental pollution, and hence the reduction of vehicle weight is one of the most effective ways of reducing overall fuel consumption and the pollutant emission [5]. Specifically, the leaf spring is one of the potential items for weight reduction in automobiles, as its weight accounts for 10–20% of the un-sprung weight (the weight which is not supported by the suspension system) of the automobile [1,3,6]. It is noted that the leaf spring is a reliable elastic component in the automobile suspension, as shown in Figure 1a, which not only suffers rigorous working conditions, but also affects the performance of the vehicle [6,7]. The reduction of un-sprung weight could significantly decrease the fatigue stress induced in the leaf spring. Therefore, the lightweight design of the leaf spring is of great concern in the automobile industry [1,8,9,10]. The fiber-reinforced polymer (FRP) composite possesses outstanding properties such as high stiffness and strength, low specific mass, and corrosion resistance, in combination with a flexible design, which has emerged as the viable candidate for the weight reduction applications without losing performance in terms of load-carrying capacity or stiffness [11,12,13,14,15,16,17,18,19,20,21]. Therefore, the introduction of composite materials enables the weight reduction in the leaf spring, which leads to a better riding performance of the vehicle as well as the reduction in vehicle cost [22].

In practice, the structure of the leaf spring’s main body used in automobiles is mainly categorized into four types, including that with constant width and thickness [23], constant width and variable thickness [24], constant thickness and variable width [6], and variable width and variable thickness [6,25]. Specifically, the leaf spring with constant width and thickness is easy to fabricate but it is weak in the thin middle part. A recent experimental and finite element (FE) analysis of such composite leaf spring showed that the weight of a glass fiber-reinforced polymer (GFRP) composite leaf spring was reduced by up to 85% of the steel leaf spring, but its maximum stress was much lower than the steel one [26]. Meanwhile, a GFRP composite leaf spring with constant width and variable thickness was designed, which also has better weight reduction and a higher nominal shear stress than the steel leaf spring [27]. However, as its shape is irregular and the difference of thickness between the middle and the end sections is large, it is not suitable for the structural connection in automobiles. A similar problem also exists in the leaf spring with constant thickness and variable width, as the difference of width between the middle and the end sections is large. Comparatively, the leaf spring with variable width and variable thickness possesses distinctive structural characteristics compared with the three previous types, where the thicker middle section is not easy to fracture and the thinner side section is easy to be connected, which is beneficial to the load-bearing capacity, the connection, and installation. A typical structure of a composite leaf spring with variable width and variable thickness is shown in Figure 1b. So far, there are few investigations about the composite leaf spring with variable width and variable thickness. Meanwhile, the structural design of such composite leaf spring based on the design concept of stiffness and material strength is still not clear. Therefore, further research is required on the optimum structural design of the composite leaf spring with variable width and variable thickness to achieve a better weight reduction.

The design of spring body structure is one of the most important problems in the design theory of composite leaf spring, as the body structure primarily determines the weight, the stress distribution state, the shape of the mold cavity, and the ply scheme framework of the spring, and hence it directly affects the performance and the manufacturing cost of the composite leaf spring. Extensive efforts have been made on the structural design and optimization of the composite leaf spring body using genetic algorithm (GA) and FE approaches [22,25,28,29,30,31,32,33]. Specifically, a GA was used to optimize the dimensions of a glass fiber-reinforced epoxy composite leaf spring with variable width, variable thickness, and equal cross-section. In comparison with the steel counterpart, the designed composite leaf spring possesses a 76% weight reduction and a 41% reduction in the maximum allowable stress, but the stiffness remains unchanged [22]. Meanwhile, a GA was applied to improve the fatigue reliability design of composite leaf springs [31]. The ply scheme of E-glass fiber/polyurethane composite leaf spring was optimized by GA, and the fatigue life was tested using a fatigue bench experiment. It is shown that the fatigue life of the designed leaf spring increased from 50,000 times to more than 540,000 times, while its stiffness was not degraded [31]. Apart from the GA, the FE approach is also used in the structural design of the composite spring. Notably, a composite leaf spring for a solar-powered light vehicle was designed by FE, and the maximum stress, deflection, and the stiffness met the design requirements of the composite leaf spring [32]. Meanwhile, a composite leaf spring made from glass fiber with epoxy resin was designed and optimized using the FE approach, and the optimized composite leaf spring possessed much lower stresses and 80% lower spring weight compared with the steel spring [25]. These previous studies have provided valuable information on the structural design of the composite leaf spring using GA and FE approaches, but the design method of composite leaf spring with variable width and variable thickness is still not fully investigated, and requires a more comprehensive investigation.

In this paper, we develop an algorithm for the structural design of the composite leaf spring with variable width and variable thickness, which is validated by comparing the experimental and FE measurements with available design requirements. Here, the GFRP composite leaf spring is investigated, as it has been widely used in the automobile plate spring. This paper starts with the development of the computational algorithm, which is used to determine the dimensions of body structure of the GFRP composite leaf spring. The composite leaf spring samples were prepared using the filament winding process and tested using the three-point bending method. The bending stiffness of the leaf spring was compared with the FE result to validate the developed algorithm. Furthermore, the stress distribution of the designed composite leaf spring under full and limit loading conditions was analyzed using the FE method. This study presents an important step for developing a sophisticated computational algorithm for the structural design of the composite leaf spring with variable width and variable thickness, which contributes to the reduction of vehicle weight.

## 2. Design of Composite Leaf Spring

In this section, we go through the requirements of structural design of the composite leaf spring. On the basis of the design theory, we present the development of a MATLAB algorithm used for the structural design.

### 2.1. Requirements of Composite Structural Design

**Figure 2:**
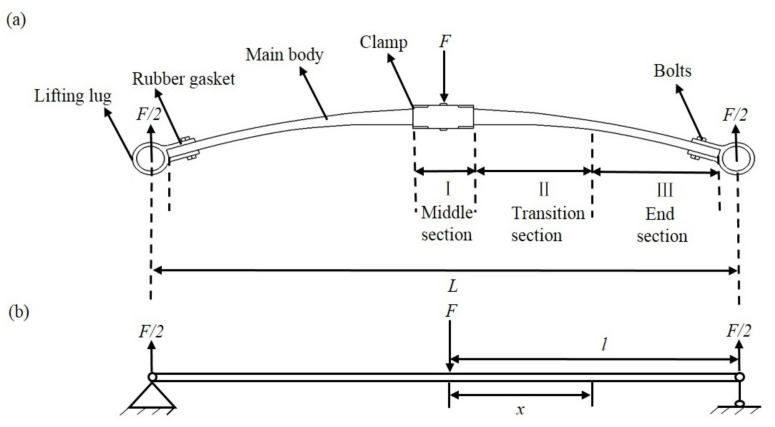
(**a**) Schematic diagram and (**b**) simplified force model of the composite leaf spring.

The composite leaf spring consists of main body, lifting lug, and clamp, while the body structure is made up of the middle section, two end sections, and the transition section between them, as shown in Figure 2a. The width of the middle and two end sections is the same, and the thickness within each of these sections is constant, while the transition section possesses variable width and variable thickness, and the cross-section area of all the sections is constant. In order to achieve the lightweight design, it requires that the used composite material is the least while ensuring the functionality and durability. The equal stress principle assumes that the ultimate stress of the shaft section of the designed leaf spring is the same, so the variation of mechanical properties of the composite is small. In the structural design of such leaf spring with variable width and variable thickness, several simplifications are adopted. Specifically, as the fiber in composite obtained from the winding forming process is basically oriented along the length direction, it is reasonable to assume that there is no variation in the composite mechanical property along the thickness and width directions. Hence, the leaf spring can be considered as transversely isotropic. Meanwhile, for the real structure of the leaf spring, it is a parabolic form, and the stiffness is considered as constant when it is deformed [29]. Therefore, the leaf spring is simplified as a cantilever beam, as shown in Figure 2. When the leaf spring is under loading, the middle section is subject to applied load *F*, and the fixed part at both ends are subject to supporting force *F*/2, as shown in Figure 2a. Comparatively, for the cantilever beam, the loading scheme is consistent with that of the leaf spring, as shown in Figure 2b. Due to the symmetric structure of the composite leaf spring, half of the cantilever beam model is selected for the calculation, with a length, *l*.

When the leaf spring works, the middle section is under applied load *F*, and the fixed parts at both ends are under supporting force *F/*2 respectively, as shown in Figure 2a. Under the applied load *F*, the stress of cantilever beam for the leaf spring is defined as,

(1) $$[\sigma ]=\frac{Fxh}{4I}$$

where [*σ*] is the allowable stress of the cantilever beam, i.e., the maximum allowable value of stress under applied load, *x* is the distance from cross-section to the middle section, *h* is the thickness of section, and *I* is the section moment of inertia, as defined in Equation (2):

(2) $$I=\frac{1}{12}bh^{3}=\frac{1}{12}sh^{2}$$

where *b* is the width, *s* is cross-sectional area, and hence, Equation (1) is transformed to:

(3) $$[\sigma ]=\frac{3Fx}{sh}$$

Meanwhile, the thickness, *h*, is related to the distance, *x*,

(4) $$h=\frac{3Fx}{s[\sigma ]}=ax$$

where *a* is defined as the changing rate of cross-sectional thickness, which represents the linear relationship of the thickness, *h*, and the distance from cross-section to the middle section, *x*. It is learned that when the load *F*, cross-section area *S*, and allowable stress [*σ*] are constant, the section thickness *h* is linear with *x* of the beam.

Apart from the strength analysis, the bending deformation of the cantilever beam under the applied load is represented by the curvature of neutral surface *ρ*:

(5) $$\frac{1}{\rho }=\frac{M(x)}{EI}$$

where *E* is the modulus of elasticity, and *M*(*x*) is the bending moment, as given by:

(6) $$M(x)=\frac{F}{2}x$$

For a segment with a length *dx*, the angle *θ* caused by the bending moment *M*(*x*) is:

(7) $$\theta \approx \tan\theta =\frac{dx}{\rho }=\frac{M(x)}{EI}dx$$

Accordingly, the deflection, *dw*, caused by displacement of the segment is:

(8) $$dw=\theta (l-x)\sin\theta \approx \theta (l-x)=\frac{M(x)}{EI}(l-x)dx$$

By integrating the deflection of the whole beam according to Equation (8), the deflection of plate spring, *w*, is obtained:

(9) $$w=\int_{0}^{l}dw=\int_{0}^{l}\frac{M(x)}{EI}(l-x)dx$$

Therefore, the bending stiffness, *k*, of the cantilever beam is obtained:

(10) $$k=\frac{F}{w}=\frac{6ashE}{\int_{0}^{l}(l-x)dx}$$

In the ride comfort requirement of the whole vehicle, the test speed of city buses is 30 km·h^−1^, and the ride comfort equivalent mean value of ordinary suspension *L*_eq_ is used as the evaluation index of ride comfort, which is no more than 115.0 dB [34]. The structure size and stiffness of the composite leaf spring affect the ride comfort of the automobile and the value of *L*_eq_. Accordingly, the design requirement of the composite leaf spring is proposed, as summarized in Table 1. Specifically, the maximum mass of designed composite leaf spring *M*_max_ is selected as 13 kg for the weight reduction design object, which is less than weight of the steel leaf spring, 17.78 kg [35]. The bending stiffness *k* should meet the required value of 124 ± 12 N·mm^−1^ based on the design parameters for the steel leaf spring [36], which are higher than that of the steel leaf spring with a value of 97 N·mm^−1^. The full load, *F*_1_, is the load on the leaf spring when the car is fully loaded in a stationary state, which is selected as 10,912 N, and it is higher than the full load of the composite leaf spring studied in a previous study with a value of 10,000 N [37]. Meanwhile, the limit load, *F*_2_, is the maximum load that the leaf spring can bear, which is selected as 23,312 N, and it is higher than the overall load of the steel leaf spring for heavy-duty vehicles with a value of 20,000 N [38]. The safety coefficient *N* reflects the degree of structural safety in the engineering structural design method, which is according to the previous work on the structure design of composite leaf spring in automobiles [39]. Specifically, when the safety factor of GFRP material is about 1.3, the reliability of GFRP material structure reaches 0.99 [40], which indicates the low failure probability of 0.01. Therefore, the safety coefficient *N* is selected with a value of 1.3. The span of the composite leaf spring, *L*, is the distance between two centers of lifting lugs with a value of 1388 mm, which refers to the size and data between various parts of the car, as shown in Figure 2a. The maximum cross-sectional area, *S*_max_, is selected as 2300 mm^2^, which is smaller than the specified value of the steel leaf spring of 2400 mm^2^ [35]. The maximum width, *b*_max_, refers to the spring width of the basic spring parameters in a range of 90–95 mm, which is selected as 92 mm [41]. The maximum thickness *h*_max_ is selected as 40 mm, which is smaller than the total thickness of the steel leaf spring with a value of 48 mm [35]. If appropriate strength [*σ*] which meets the strength design requirement and cross-sectional area, *S*, which meets the design requirement of leaf spring are selected in the design process, the bending stiffness, *k*, should meet the required value of 124 ± 12 N·mm^−1^. According to Table 1, the limit load of half of the simplified leaf spring model is: 23,312/2 N = 11,656 N, which is the maximum load of the leaf spring.

Apart from the stiffness requirement, the composite leaf spring should meet the strength design requirement. In this study, when the leaf spring works, it is compressed along the vertical direction, and hence the compressive strength is considered as the material strength limit, *σ*, in the design of the composite leaf spring. The compressive strength of the composite material has a value of 875 MPa, which is adopted from E-glass fiber/epoxy composite material with 40% volume fraction of fiber, i.e., the experimental sample used in this study, as introduced subsequently. For a specific component composed of a certain material, the maximum allowable value of working stress is called allowable stress [*σ*]. The allowable stress has a relationship with limit stress, *σ*_u_, as: [*σ*] = *σ*_u_/*N*, where the ultimate strength of material is called limit stress, *σ*_u_, and *N* is the safety factor. The maximum stress of the composite leaf spring under loading should not exceed the allowable stress of the material. In consideration of the safety factor of 1.3 as listed in Table 1, the allowable stress [*σ*] of composite leaf spring is determined as: [*σ*] = 875/1.3 MPa = 673 MPa.

### 2.2. Structure Design of Composite Leaf Spring

**Figure 3:**
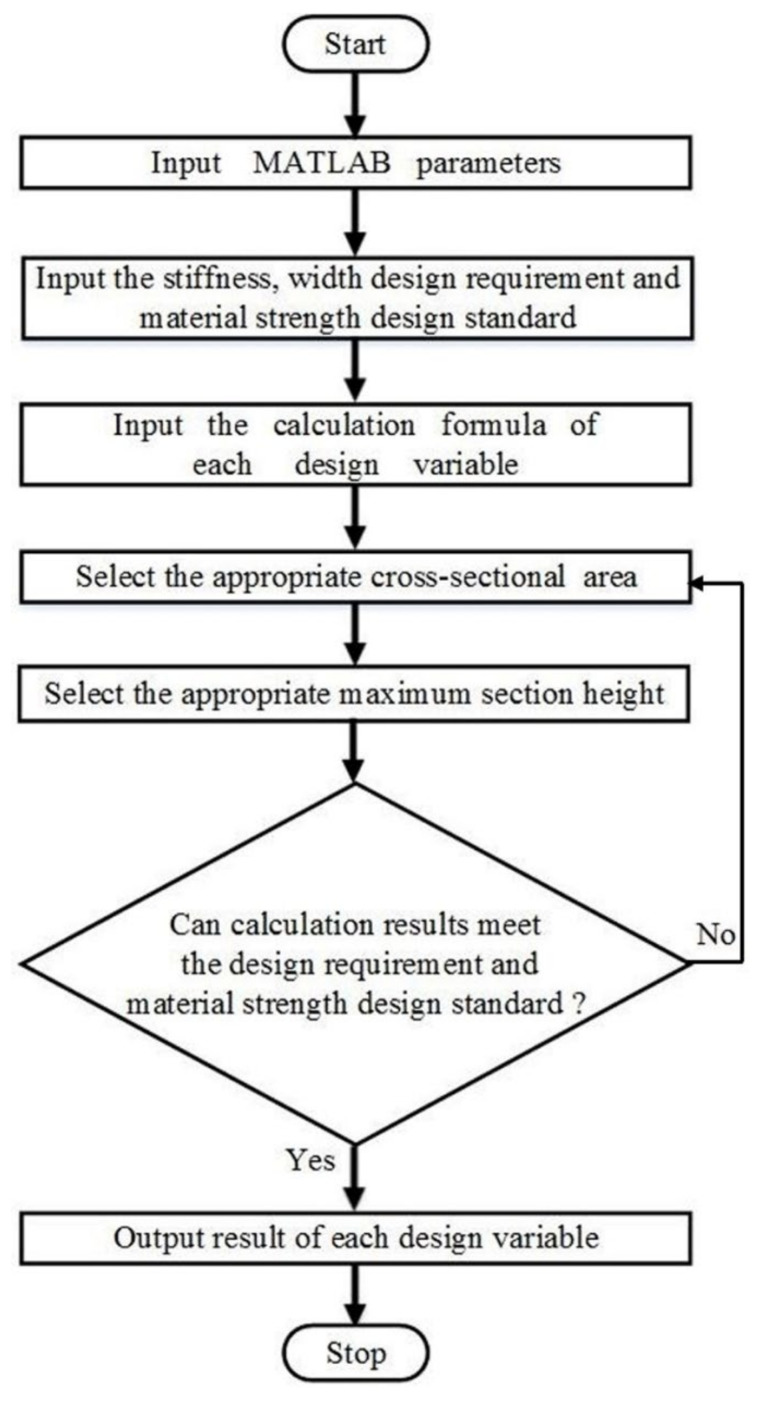
MATLAB calculation flow chart for structure design of the composite leaf spring.

To meet the design requirements, the structure of the composite leaf spring should be designed carefully. Notably, the span, *L*, has a fixed value, and the cross-sectional area S, width *b*, and thickness *h* of the leaf spring are to be determined. The middle part of the leaf spring is connected and fixed by the clamp and the two ends are fixed by the lifting lug, so the middle part and the two ends are the zones with equal width and thickness, while the transition section is the zone with variable width and variable thickness. Here, MATLAB software is used for the structural design of the leaf spring. The calculation flow chart adopted in MATLAB is shown in Figure 3. Firstly, the parameters of the composite leaf spring are inputted in MATLAB. Specifically, the cantilever length, *l*, equals half of span, *L*, with a value of 694 mm. The elastic modulus *E* along length direction has a value of 52,500 MPa, which is adopted from the experimental sample, as introduced in the Appendix A, subsequently. The length direction coordinate of cantilever beam *i* is defined as 0, which indicates the coordinate origin. The increment along length direction *dx*, thickness direction *dh*, and sectional area *dS* is defined as 1 mm, 0.1 mm, and 1 mm^2^, respectively. Secondly, several requirements and standards of composite leaf spring as shown in Table 1 are inputted, including the bending stiffness requirement of 124–12 N·mm^−1^ ≤ *k* ≤ 124 + 12 N·mm^−1^, strength design standard of *σ* ≤ 673 MPa, and width requirement of *b* ≤ 92 mm. Thirdly, the calculation formula of the design variable is inputted, including section width *b*, maximum stress *σ*_max_, section thickness *h*, deflection *w*, and bending stiffness *k*, as shown in Equations (2)–(4), (9), and (10), respectively. After defining these parameters and calculation equations, the initial value of the cross-sectional area, *S*, and the maximum section thickness, *h*_max_, are selected from the range of 1500 to 2300 mm^2^ and 25 to 40 mm randomly, where the maximum value is defined in Table 1. Using the initial values of *S* and *h*_max_, the design variables of the composite leaf spring are calculated using the program in MATLAB software according to the calculation principle as described in Section 2.1, including maximum section width *b*_max_, and maximum deflection *w*_max_. According to these calculated design variables, the bending stiffness, *k*, and maximum stress, *σ*_max_, under loading are calculated based on Equations (10) and (3) respectively, which are compared with the design requirement of stiffness and strength of composite leaf spring, as summarized in Table 1. It is learned from Equation (10) that the stiffness, *k*, increases with the increasing cross-sectional area *S* and increasing thickness *h*. If the stiffness result is larger than the design requirement, another set of a smaller *S* and *h*_max_ is selected automatically in the MATLAB algorithm from the range as described previously, with the difference of *dS* and *dh*, respectively. Comparatively, if the stiffness result is smaller than the design requirement, another set of a larger *S* and *h*_max_ is selected. Based on each determined set of *S* and *h*_max_, the calculation results are compared with the design requirements of the leaf spring. Because the change range of stiffness design requirement is small, the design principle is that the *σ*_max_ under loading reaches the highest value on the premise that the *k* meets the design requirements. After multiple screening and calculation, the set of *S* and *h*_max_ was determined when the calculation results meet the design requirement.

### 2.3. Designed Structure of Composite Leaf Spring

When the cross-sectional area, *S*, is 2000 mm^2^ and the maximum thickness *h*_max_ is 37.1 mm, the calculation results of the composite leaf spring meet the design requirement, as listed in Table 1. The resulted structural characteristics of the leaf spring are shown in Table 2. Specifically, the length of transition section, *l*_t_, is 248 mm. The maximum width, *b*_max_**, is 90 mm, which is the width of the middle section of the main body. The maximum deflection, *w*max, is 180 mm when the leaf spring is under the limit load, *F*_2_. The bending stiffness of the composite leaf spring is *k* = *F*/*w* = *F*_2_/*w*_max_ = 23.312/180 N·mm^−1^ = 129.5 N·mm^−1^. The maximum stress, *σ*_max_, along the length direction of the composite leaf spring is 580 MPa. According to the comparison of calculated *k* and *σ*_max_, it is observed that the calculated *σ*_max_ is much smaller than the design requirement. Extra optimization is carried out to confirm whether there is a better set of the *k* and *σ*_max_. Accordingly, another set of calculation results obtained from multiple screening and calculation in MATLAB were investigated. It was determined that for the original calculation result, the composite leaf spring possessed the higher *k* and *σ*_max_ compared with the other solution. Therefore, it is indicated that the presented result is the optimal one.

## 3. Experimental and Finite Element Analysis of Designed Composite Leaf Spring

In this section, we present the experimental and simulation details for evaluations of the mechanical behavior of the composite leaf spring with the designed structure, so as to verify the rationality of the developed MATLAB algorithm.

### 3.1. Experiment of Composite Leaf Spring

**Figure 4:**
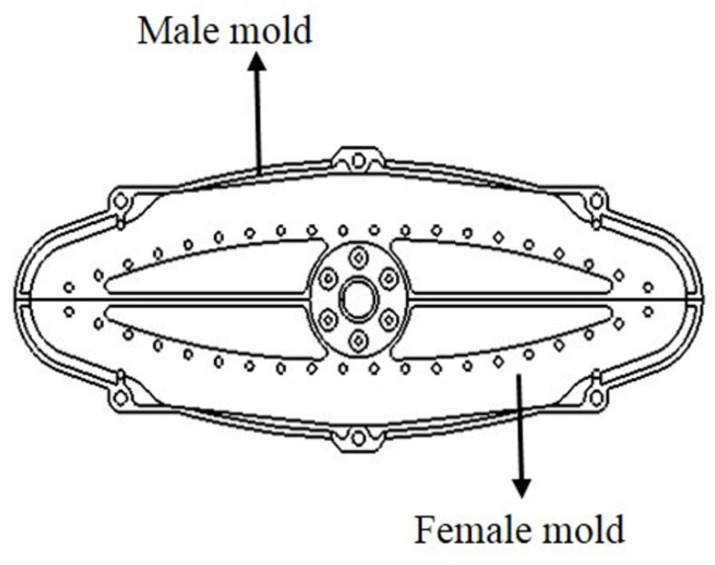
Winding mold of composite leaf spring.

Based on the design parameters, the GFRP composite leaf spring was prepared and measured using the three-point bending test. In this work, the glass fiber is used in manufacturing the FRP composite instead of carbon fiber as its cost is comparatively lower. Meanwhile, the mechanical performance of GFRP meets the requirements of composite material in the automobile industry, and the GFRP is widely used for composite structure in actual engineering applications [42]. Therefore, the glass fiber is considered in this work. E-glass fiber/epoxy composite material with 40% volume fraction of fiber was taken to manufacture the composite leaf spring samples. The E-glass fiber was chosen as the reinforcement material. The tensile strength and modulus of E-glass fiber are 2.482 and 82 GPa, as provided by the supplier Taishan Glass Fiber Company. The epoxy used here was composed of two parts, including epoxy resin and anhydride-modified curing agent, which was supplied by Tianjin Dasen Company. The GFRP composite leaf spring was manufactured using the filament winding process, as it effectively reduces the processing cost of composites [43,44,45,46]. Specifically, the winding mold of the composite leaf spring includes two parts, i.e., the male mold and the female mold, as shown in Figure 4. The winding mold is a symmetrical geometric structure with variable thickness and variable width. Two leaf springs can be obtained by the winding process. The winding parameters are set during the winding process control, mainly including the winding tension and winding speed. The number of winding layers of the composite leaf spring was determined first, which was obtained by dividing the maximum thickness of the composite leaf spring of 37.1 mm (Table 2) by the thickness of a single composite layer of 3.3 mm obtained by measuring the sample before the winding process. Accordingly, the total number of winding layers in this work was determined as eleven. During the manufacturing process, the polymer matrix was firstly prepared, where the epoxy resin and curing agent were mixed with the mass ratio of 100:84.5 and stirred evenly, which were then kept at a constant temperature of 50 °C for 1 h for the sufficient cross-linking reaction between epoxy resin and curing agent. After that, the winding process of the composite leaf spring was carried out. The winding parameters are set during the winding process control, mainly including the winding tension and winding speed. Specifically, the winding tension is required to decrease every few layers, so as to reduce the pressure of the outer layer on the inner layer and to avoid the fiber buckling. Meanwhile, the winding speed was controlled according to the rotation rate of the core mold of the winding mold. It was determined that with the decreasing tension from 60, 40, to 20 N, and the winding speed of 3 r·min^−1^, the manufactured composite leaf spring possessed the higher fiber volume fraction of 65.7% compared with the other two cases of 70, 50, to 30 N, and 50, 30, to 10 N. Therefore, the winding tension decreasing from 60, 40, to 20 N and the winding tension speed of 3 r·min^−1^ were selected in this work. During the winding process, four layers of fiber after immersing in the glue were wound around the whole winding mold layer-by-layer under tension of 60 N, another four layers were wound under 40 N, and a final three layers were wound around under 20 N. After the filament winding process, the sample was cured at the temperature level of 85 °C for 0.5 h, 95 °C for 1 h, 105 °C for 0.5 h, and 120 °C for 1 h, respectively. Subsequently, it was naturally cooled down to room temperature for pressure relief and demolding. Three glass fiber-reinforced polymer (GFRP) composite leaf spring samples were prepared. The overall dimensions of the spring samples were 1388 × 90 × 37 mm, as adopted from the design results listed in Table 2. The technological parameters of the filament winding process are summarized in Table 3. After the above winding parameters are set, the winding machine is started, and the leaf spring is wound according to the set winding process. Through the winding process control system, the fibers are aligned along the beam axis.

**Figure 5:**
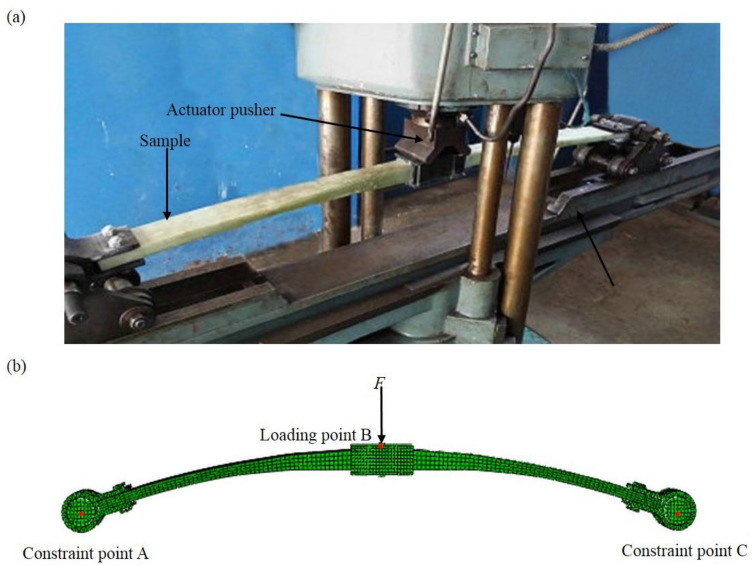
(**a**) A photograph of the test rig and (**b**) finite element (FE) model of the three-point bending test of composite leaf spring: the motion constraint of the joints is realized by constraining the freedom degree of the point A and C at the two ends.

According to the prepared composite leaf spring, the mass of each section was measured. Specifically, the mass of the main body, lifting lug and its bolts, clamp and its bolts, and gasket were measured as 6.40, 1.88, 1.36, and 0.11 kg, respectively. Therefore, the total mass of the spring samples was 9.75 kg, which meets the design requirement as listed in Table 1. The material properties of the composite leaf spring were obtained by following the experimental procedures described by relevant national standards [47,48,49,50]. The bending stiffness of the composite leaf spring sample was tested using the three-point bending method, as shown in Figure 5a. The clamp section of the spring sample was loaded vertically by the actuator pusher. The load was applied under a crosshead force control at a rate of 1000 N·s^−1^. The loading process was about 20 s long. The load was gradually increased from 0 to 21.8 kN to measure the mid-span deflection. During the experiment, the load and the deflection of the GFRP composite leaf spring were recorded.

### 3.2. Finite Element Analysis of Composite Leaf Spring

In order to further study the mechanical properties of the composite leaf spring in the structural design, the composite leaf spring was modeled and investigated using the finite element method. Based on the design result as shown in Table 2, the three-dimensional FE model of the composite leaf spring was created, as shown in Figure 5b. In the three-point bending test, the translation and the rotations at the spring ends in the test ring are not free, while the translation along the span direction and the rotation along the width direction of composite leaf spring are free. Comparatively, in FE analysis, the freedom degrees of point A and C at the two ends are constrained in the FE model, while the displacement along the span direction and the rotation along the width direction of the composite leaf spring are free, which are equivalent to the conditions in the three-point bending test. The main body of the leaf spring is formed by multi-section curved surface, and the lifting lug and clamp are formed by plane stretching. After that, the mechanical properties of the leaf spring model are defined, including Young’s modulus and Poisson ratio, which are adopted from E-glass fiber/epoxy composite material with 40% volume fraction of fiber. Mechanical property parameters include tensile modulus (*E*_1_, *E*_2_, *E*_3_), shear modulus (*G*_12_, *G*_13_, *G*_23_), Poisson’s ratio (*μ*_12_, *μ*_13_, *μ*_23_), tensile strength (*X*_1_, *X*_2_, *X*_3_), and compressive strength (*Y*_1_, *Y*_2_, *Y*_3_) along length, thickness, and width directions, and interlaminar shear strength, *σ*_ILSS_, and longitudinal and transverse shear strength, *σ*_τ±45°_. Notably, *σ*_ILSS_ is the ultimate strength of samples under pure shear load, which equals the resultant force of shear force along thickness-width direction and length-thickness direction. *σ*_τ±45°_ is ultimate shear stress of single-layer fiber-reinforced composites under normal axial longitudinal and transverse pure shear loads, which equals the shear strength along length-width direction. The detailed procedure of the parameter measurement is described in Appendix A. The results of these parameters are shown in Table 4.

Based on the designed composite leaf spring model, the mass of each part is calculated. The volume of each part is obtained by measuring the three-dimensional model, as shown in Figure 5b. Accordingly, the main body is made of GFRP material, with the density of 2.14 × 10^3^ kg·m^−3^ and the volume of 3.00 × 10^−3^ m^3^, so the mass of the main body is 6.42 kg. The lifting lug and its bolts are made of aluminum, with the density of 2.70 × 10^3^ kg·m^−3^ and the volume of 0.69 × 10^−3^ m^3^, so the mass of the lifting lug and its bolts is 1.86 kg. The clamp and its bolts are made of steel, with the density of 7.89 × 10^3^ kg·m^−3^ and the volume of 0.17 × 10^−3^ m^3^, so the mass of the clamp and its bolts is 1.34 kg. Meanwhile, the gasket is made of rubber, with the density of 1.00 × 10^3^ kg·m^−3^ and the volume of 0.10 × 10^−3^ m^3^, so the mass of the gasket is 0.10 kg. Therefore, the total mass of the composite leaf spring is calculated as 9.72 kg, which is very close to the mass of the composite leaf spring samples (9.75 kg) as presented in the previous section, and both values meet the design requirements, as listed in Table 1. Comparatively, the mass of the steel leaf spring with the same volume is 31.24 kg, and hence the weight reduction of the composite leaf spring is 69%.

Based on the FE model, stiffness and stress state of the composite leaf spring are analyzed. The FE model is meshed before loading. In the FE calculation, the mesh size was selected from 1.0, 2.0, 3.0, 4.0, 5.0, 6.0, and 7.0 mm. For the leaf spring, the maximum deflection, *w*_max_, under the limit loading condition is an important parameter for comparison. The calculation time of FE analysis was also counted for comparison. It is measured that when the mesh size is less than 4.0 mm, the change of maximum deflection, *w*_max_, is very small with the mesh size, but the calculation time increases significantly. In consideration of the result convergence and the calculation efficiency, the mesh size of 4.0 mm is selected for the FE modelling. Considering the structure of the composite leaf spring, body element C3D8I is used to discretize the composite leaf spring, as it can avoid the shear locking phenomenon. In order to ensure the mesh quality, hexahedron is used for meshing. The model includes 16 contact surfaces and 74,327 hexahedral linear reduction elements. During the FE analysis of stiffness and stress state of the composite leaf spring, the following boundary conditions are used: the center of the leaf spring along the length direction is kept fixed, and the movement of the leaf spring along the width and thickness directions are fixed at both ends. The stiffness of the composite leaf spring model was tested using the three-point bending method, as shown in Figure 5b. The load, *F*, is applied to the middle point B, and the motion constraint of the joints is realized by constraining the freedom degree of the points A and C at the two ends. In the three-point bending test, the horizontal displacements in the test ring and the rotations at the spring ends are not free, while the translation along the span direction and rotation along the width direction of the composite leaf spring are free. Accordingly, in FE analysis, the horizontal displacements in the leaf spring and the rotations at the spring ends are not free, while the displacement along span direction and the rotation along width direction of the composite leaf spring are free, as shown in Figure 5b. Therefore, the constraints between the three-point bending test ring and the FE model are equivalent. The setup and loading speed under force control were the same as in Section 3.1. The load gradually increased from 0 to 21.8 kN (*F*_max_), and the maximum deformation of leaf spring was measured as *w*_max_. According to the measurement, the bending stiffness of leaf spring, *k*, was measured as *F*_max_ /*w*_max_. Apart from the stiffness performance, the strength performance of the composite leaf spring was studied. The stress distribution of the composite leaf spring under full loading condition and limit loading condition was studied. The loading area, *S*_0_, of clamp in the middle section was measured from the FE model as 0.011 m^2^. Under full and limit loading conditions of constant force, *F*_1_ and *F*_2_, as listed in Table 1, the value of distributed force was calculated as *F*/*S*_0_, with a value of 99,272 N·mm^−2^ and 2,119,272 N·mm^−2^, respectively. During the loading process, the deflection of each node was read in FE analysis, and the deflection distribution of the composite leaf spring was obtained. During the loading process, the stress state of each node was read in FE analysis, and the stress along each direction of the composite leaf spring was obtained.

Based on the FE model, stiffness and stress state of the composite leaf spring are analyzed. The FE model is meshed before loading. In the FE calculation, the mesh size was selected from 1.0, 2.0, 3.0, 4.0, 5.0, 6.0, and 7.0 mm. For the leaf spring, the maximum deflection, *w*_max_, under the limit loading condition is an important parameter for comparison. The calculation time of FE analysis was also counted for comparison. It is measured that when the mesh size is less than 4.0 mm, the change of maximum deflection, *w*_max_, is very small with the mesh size, but the calculation time increases significantly. In consideration of the result convergence and the calculation efficiency, the mesh size of 4.0 mm is selected for the FE modelling. Considering the structure of the composite leaf spring, body element C3D8I is used to discretize the composite leaf spring, as it can avoid the shear locking phenomenon. In order to ensure the mesh quality, hexahedron is used for meshing. The model includes 16 contact surfaces and 74,327 hexahedral linear reduction elements. During the FE analysis of stiffness and stress state of the composite leaf spring, the following boundary conditions are used: the center of the leaf spring along the length direction is kept fixed, and the movement of the leaf spring along the width and thickness directions are fixed at both ends. The stiffness of the composite leaf spring model was tested using the three-point bending method, as shown in Figure 5b. The load, *F*, is applied to the middle point B, and the motion constraint of the joints is realized by constraining the freedom degree of the points A and C at the two ends. In the three-point bending test, the horizontal displacements in the test ring and the rotations at the spring ends are not free, while the translation along the span direction and rotation along the width direction of the composite leaf spring are free. Accordingly, in FE analysis, the horizontal displacements in the leaf spring and the rotations at the spring ends are not free, while the displacement along span direction and the rotation along width direction of the composite leaf spring are free, as shown in Figure 5b. Therefore, the constraints between the three-point bending test ring and the FE model are equivalent. The setup and loading speed under force control were the same as in Section 3.1. The load gradually increased from 0 to 21.8 kN (*F*_max_), and the maximum deformation of leaf spring was measured as *w*_max_. According to the measurement, the bending stiffness of leaf spring, *k*, was measured as *F*_max_ /*w*_max_. Apart from the stiffness performance, the strength performance of the composite leaf spring was studied. The stress distribution of the composite leaf spring under full loading condition and limit loading condition was studied. The loading area, *S*_0_, of clamp in the middle section was measured from the FE model as 0.011 m^2^. Under full and limit loading conditions of constant force, *F*_1_ and *F*_2_, as listed in Table 1, the value of distributed force was calculated as *F*/*S*_0_, with a value of 99,272 N·mm^−2^ and 2,119,272 N·mm^−2^, respectively. During the loading process, the deflection of each node was read in FE analysis, and the deflection distribution of the composite leaf spring was obtained. During the loading process, the stress state of each node was read in FE analysis, and the stress along each direction of the composite leaf spring was obtained.

## 4. Results and Discussion

### 4.1. Experimental Analysis of Composite Leaf Spring

**Figure 6:**
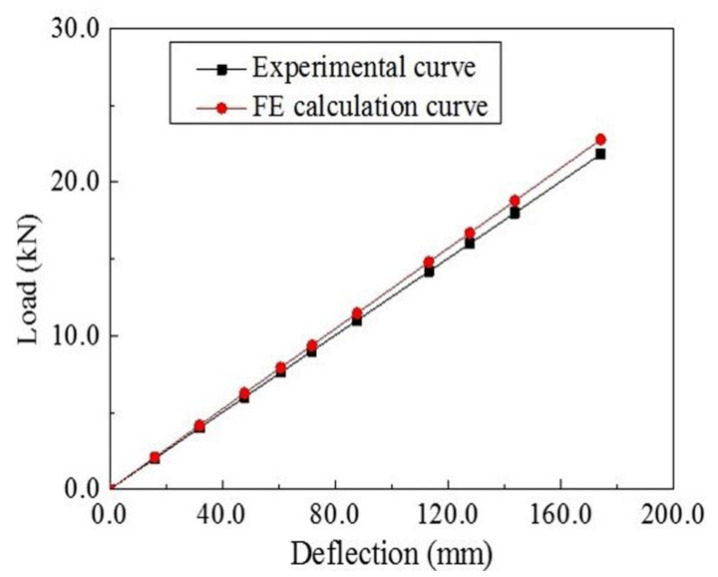
The load-deflection curves of the composite leaf spring under the three-point bending test and FE simulation.

According to the three-point bending test, the load-deflection curve of the composite leaf spring sample was obtained, as shown in Figure 6. By performing the linear regression of the curve, it is measured that the bending stiffness of the leaf spring is 125.3 N·mm^−1^, which shows a good agreement with the design requirement of 124 ± 12 N·mm^−1^, as listed in Table 1. The close agreement demonstrates the applicability of the developed MATLAB algorithm in the structural design of the composite leaf spring.

### 4.2. Finite Element Analysis of Composite Leaf Spring

**Figure 7:**
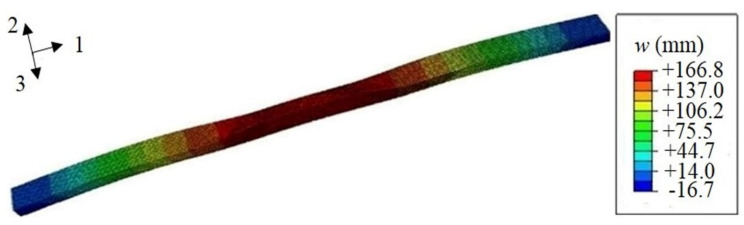
The deflection distribution diagram obtained from the FE simulation, where 1, 2, and 3 in the axis denote the length, thickness, and width direction of the composite leaf spring, respectively.

The load-deflection curve obtained from FE simulation is shown in Figure 6. The FE result is very close to the experimental curve, which indicates the agreement with the experiment. The deflection distribution diagram of the FE composite leaf spring model under the three-point bending test is shown in Figure 7. The maximum deflection is *w*_max_ = 166.8 mm, and the maximum force, *F*_max_, is 21,800 N according to Section 3.2, so the bending stiffness is *k* = *F*_max_/*w*_max_ = 21,800/166.8 N·mm^−1^ = 130.7 N·mm^−1^, which is close to the experimental result with a value of 125.3 N·mm^−1^, as presented previously. It is observed that both experimental and FE results meet the bending stiffness design requirement of 124 ± 12 N·mm^−1^ (see Table 1).

**Figure 8:**
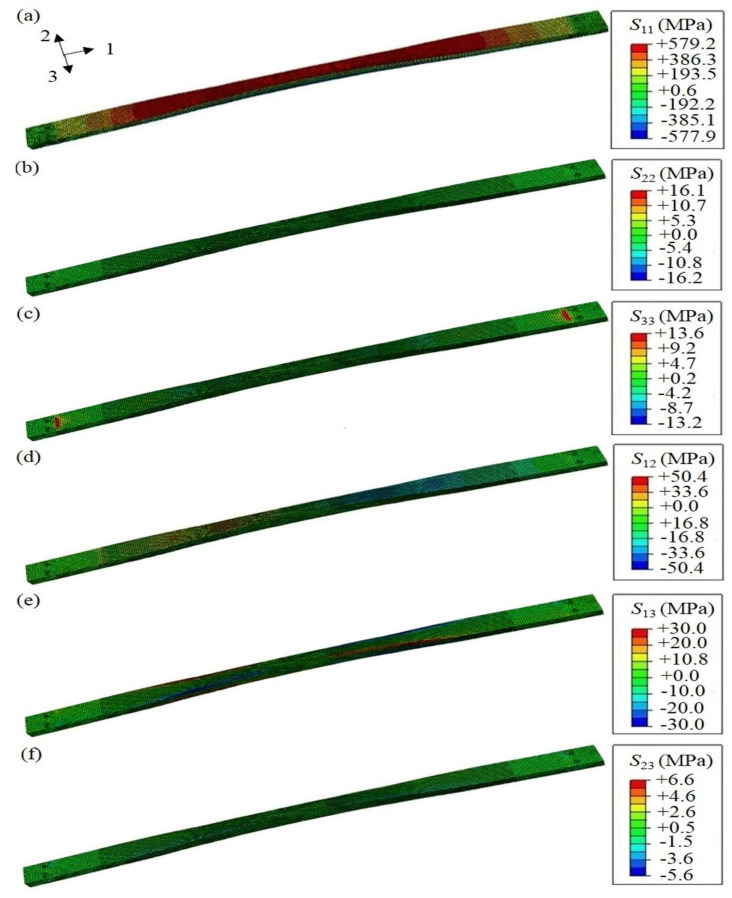
Stress distribution along all directions of the composite leaf spring under full loading condition: normal stress of (**a**) length direction *S*_11_, (**b**) thickness direction *S*_22_, and (**c**) width direction *S*_33_; shear stress of (**d**) length-thickness direction *S*_12_, (**e**) length-width direction *S*_13_, and (**f**) thickness-width direction *S*_23_.

The mechanical behavior of the FE composite leaf spring model under the distributed force on the middle section is presented here. Under full loading condition, the stress distribution of the composite leaf spring along each direction is calculated, as shown in Figure 8. The FE results of the composite leaf spring under full loading condition are summarized in Table 4, in comparison with the material strength values of E-glass fiber/epoxy composite, which are obtained by dividing the strength value in Table 4 by the safety coefficient of 1.3. Specifically, the distribution of normal stress along length direction *S*_11_ is shown in Figure 8a, which is in the range between −577.9 to +579.2 MPa. The absolute values of these two strengths are very close. The geometric structure of the composite leaf spring sample manufactured by the winding process is symmetrical. When the sample is loaded in the three-point bending test, the upper surface of the sample is compressed, and the bottom surface of the sample is stretched. In the FE simulation, the upper surface of the composite leaf spring under loading is colored in red, as shown in the FE results, and the positive sign indicates compressive stress on the upper surface. Meanwhile, the bottom surface of the composite leaf spring under loading is colored in blue, as shown in the FE results, and the negative sign indicates tensile stress on the bottom surface. It is noted that the loading condition of the composite leaf spring model is consistent with that in the three-point bending test. In Table 5, the tensile strength along length direction is 1300 MPa, and the tensile strength value after considering the safety coefficient is 1000 MPa. As 577.9 MPa is smaller than 1000 MPa, it meets the material strength requirement. Similarly, 579.2 MPa is smaller than the compressive strength value after considering the safety coefficient with a value of 673.1 MPa, which meets the material strength requirement. The failure of the composite leaf spring is mainly along the length direction, so the strength performance along the length direction is most important. Both 577.9 and 579.2 MPa are much larger than the maximum stress, 462.2 MPa, of a composite leaf spring obtained from the optimal design by GA [22]. Meanwhile, the simulation results of 577.9 and 579.2 MPa are much larger than the tensile strength, 450 MPa, of a steel leaf spring [51]. It is shown that the composite leaf spring designed by the MATLAB algorithm in this paper has better strength performance along the length direction under full loading condition. Meanwhile, the distribution of normal stress along thickness direction, *S*_22_, and along width direction, *S*_33_, is shown in Figure 8b,c, which is in the range of −16.2 to +16.1 MPa and −13.2 to +13.6 MPa, respectively. In Table 5, the tensile strength value after considering the safety coefficient is 34.6 MPa, which is higher than the value of *S*_22_ and *S*_33_. Furthermore, the distribution of shear stress along the length-thickness direction, *S*_12_, length-width direction, *S*_13_, and thickness-width direction, *S*_23_, is shown in Figure 8d–f, respectively. Additionally, the longitudinal-transverse shear strength, *σ*_τ±45°_, equals the shear strength along the length-width direction, *S*_13_, with a value of 30.0 MPa. Meanwhile, the interlaminar shear strength, *σ*_ILSS_, is the resultant force of shear force along thickness-width direction, *S*_23_, and along length-thickness direction, *S*_12_, with a value of 50.4 MPa. It is observed that for each parameter, the calculated stress along each direction is smaller than the requirement of material strength, which demonstrates that the calculation results are reasonable.

**Figure 9:**
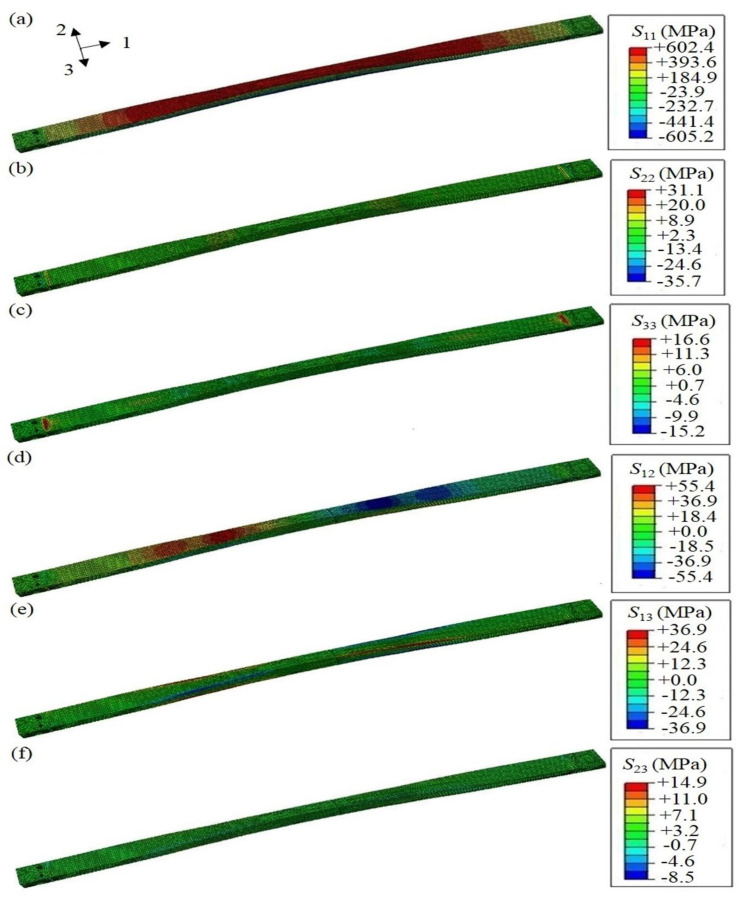
Stress distribution along all directions of the composite leaf spring under limit loading condition: normal stress of (**a**) length direction *S*_11_, (**b**) thickness direction *S*_22_, and (**c**) width direction *S*_33_; shear stress of (**d**) length-thickness direction *S*_12_, (**e**) length-width direction *S*_13_, and (**f**) thickness-width direction *S*_23_.

Apart from the full loading condition, the stress distribution of the composite leaf spring under limit loading condition is calculated, as shown in Figure 9. Specifically, the distribution of normal stress along length direction *S*_11_ is shown in Figure 9a, which is in the range between −605.2 to +602.4 MPa. The absolute values of these two strengths are very close. In Table 4, the tensile strength value after considering the safety coefficient is 1000 MPa. As 605.2 MPa is smaller than 1000 MPa, it meets the material strength requirement. Similarly, 602.4 MPa is smaller than the compressive strength value after considering a safety coefficient with a value of 673.1 MPa, which meets the material strength requirement. Both 605.2 and 602.4 MPa are much larger than the maximum stress 462.2 MPa of a composite leaf spring obtained from the optimal design by GA [22]. Meanwhile, the simulation results of 605.2 and 602.4 MPa are also much larger than the tensile strength, 450 MPa, of a steel leaf spring [51], which demonstrates that the composite leaf spring designed by the MATLAB algorithm here has better strength performance along length direction under limit loading condition. Meanwhile, the distribution of normal stress along thickness direction, *S*_22_, and along width direction, *S*_33_, is shown in Figure 9b,c, which is in the range of −35.7 to +31.1 MPa and −15.2 to +16.6 MPa, respectively. In Table 5, the tensile strength value after considering the safety coefficient is 34.6 MPa, which is higher than the values of *S*_22_ and *S*_33_. Furthermore, the distribution of shear stress along length-thickness direction *S*_12_, length-width direction *S*_13_, and thickness-width direction *S*_23_, is shown in Figure 9d–f, respectively. Additionally, the longitudinal-transverse shear strength, *σ*_τ±45°_, has a value of 36.9 MPa, and the interlaminar shear strength, *σ*_ILSS_, has a value of 57.0 MPa. As the calculated stress along each direction is smaller than the requirement of material strength, the calculation results are reasonable.

In summary, FE results of the composite leaf spring under full and limit loading conditions meet the material strength requirement. However, it is observed in Table 5 that there is still a gap between some of the maximum stresses and the allowable values, such as compressive strength along thickness and width directions of the leaf spring. Extra optimization is carried out to confirm whether there is a better set of results. It is noted that when the compressive strength along thickness and width directions of the leaf spring in Table 5 increases, other maximum stresses such as longitudinal and transverse shear strength, *σ*_τ±45°_, and interlaminar shear strength, *σ*_ILSS_, do not meet the strength requirements. It is indicated that the presented result is the optimal.

**Figure 10:**
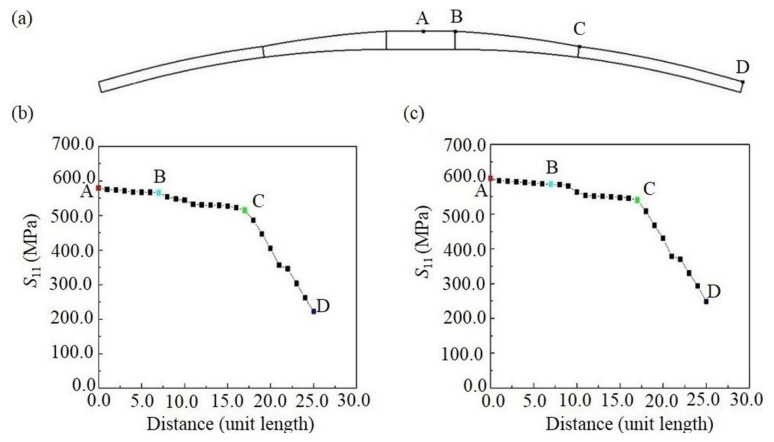
(**a**) Schematic diagram of representative nodes of composite leaf spring, and the normal stress along length direction *S*_11_ of composite leaf spring (**b**) under full loading condition and (**c**) under limit loading condition, where point A, B, C and D in the figure denotes the center, the start of the variable width section, the start and the end of the constant width section of composite leaf spring, respectively.

From previous discussion, it is learned that under two loading conditions, the composite leaf spring is mainly subject to normal stress along length direction *S*_11_. The node with the maximum stress is located in the center of the leaf spring, as shown as point A in Figure 10a. This node is defined as the coordinate origin along the length direction, and the distance between two adjacent nodes along the length direction is regarded as d*. A series of nodes along the length direction of the composite leaf spring are selected successively. Notably, point B and C as shown in Figure 10a refer to the start of the variable width section and constant width section, respectively. The normal stress, *S*_11_, of different nodes under full loading and limit loading conditions is shown in Figure 10b,c, respectively. It is observed that the tensile stress of the composite leaf spring decreases steadily along the length direction. Specifically, the decreasing rate of *S*_11_ along the variable width section BC is close to that of the middle section AB, and the decreasing rate along constant width section CD is the largest. The reason is that the slope affects stress transfer, where the greater the slope, the greater the stress changing. As the slope of the CD section is the largest, it possesses the largest decreasing rate of *S*_11_. Furthermore, as the value of limit load is larger than the full load, the stress value of the same node in Figure 10c is higher than that of Figure 10b.

### 4.3. Discussion of Structural Design of Composite Leaf Spring

In this work, the MATLAB algorithm is developed to design the structure of the composite leaf spring with variable width and variable thickness. The stiffness of the designed composite leaf spring is measured using the experimental and FE approach, which meets the design requirements as listed in Table 1. The close agreement between the measured results and the design requirements demonstrates the applicability of the MATLAB algorithm in the structural design of the composite leaf spring. However, it is noted that there are some assumptions in the development of the MATLAB algorithm. For instance, the optimal design result obtained in this paper is based on the structure of a composite leaf spring with variable width and variable thickness, which may not be applicable to other structural forms with different design and material strength requirements. Meanwhile, there is no variation in the mechanical property along the thickness and width directions of the composite leaf spring and the stiffness is considered as constant when it is deformed in the simulation process. For the composite leaf spring obtained from the winding forming process, the fiber is basically oriented along the length direction. Accordingly, the mechanical property of the leaf spring along the length direction is much better that that along the thickness and width directions, and the difference of mechanical properties along the thickness and width directions can be ignored as compared to that along the length direction. In the FE simulation, it is assumed that there is no variation of mechanical properties along the thickness and width directions of the composite leaf spring. Nevertheless, the calculated values of the composite mechanical properties meet the stiffness and strength requirements of the composite leaf spring, which demonstrates the applicability of the simplified approach in the FE simulation. In future work, the variation of the composite mechanical property along the thickness and width directions could be incorporated in the simulation process for the more accurate simulation of a composite leaf spring obtained from the winding form process. Therefore, the accuracy of the developed algorithm could be further improved by considering these material properties and performance characteristics of the leaf spring in the design work.

The actual stress characteristics of fiber depend on the manufacturing process of the composite leaf spring. According to the mid-plane stress assumption of the Classical Laminate Theory, the shear stress along the width and thickness directions of laminates is zero, and the effect of ply angle on the shear stress in the filament winding process is not considered in the Classical Laminate Theory. Therefore, it may not be accurate to predict behavior of the composite structure made by the winding process using this theory. In the isotropic beam theory, as the fiber in the composite obtained from the winding forming process is basically oriented along the length direction, it is reasonable to assume that there is no variation in the composite mechanical properties along the thickness and width directions. Hence, the leaf spring can be considered as isotropic, transversely. Therefore, the isotropic beam theory is used to predict the behavior of the composite structure in this paper. The composite leaf spring structure with unidirectional filament winding can be analyzed using the Classical Laminate Theory, which could be implemented in the MATLAB algorithm developed in this work for the structure design of unidirectional composite laminates.

For the engineering applications of the composite leaf spring, the stress distribution is very important, as it directly reflects the bearing capacity along the length, thickness, and width directions of the leaf spring under loading. In this paper, the stress distribution along the three directions of the designed composite leaf spring under full and limit loading conditions was analyzed using the FE simulation. The measured strength values along the three directions meet the material strength requirements, which indicates that the MATLAB algorithm is able to design the composite leaf spring with variable width and variable thickness. It is noted that such stress analysis under loading conditions is not generally reported in previous research of structure design of a composite leaf spring.

## 5. Conclusions

In this study, we developed the MATLAB algorithm to design the structure of a glass fiber-reinforced composite leaf spring with variable width and variable thickness, which was validated by comparing the mechanical properties of the designed composite leaf spring from experiment and FE simulation with the material design and strength requirements. Based on the calculated structural characteristics from the MATLAB algorithm, the composite leaf spring was prepared using the filament winding process, which possesses a bending stiffness of 125.3 N·mm^−1^, as measured using the three-point test. Furthermore, the three-dimensional FE model of the designed composite leaf spring is developed, which is subject to full loading and limit-loading conditions. It is shown that the strength of the composite leaf spring under full loading and limit loading conditions meet the material strength requirements. Both experimental and FE simulation results of the designed composite leaf spring meet the design requirements of the composite leaf spring, which validates the MATLAB algorithm in the structural design of the composite leaf spring. The MATLAB algorithm developed in this paper provides a reasonable and effective method for the design of the composite leaf spring with variable width and variable thickness, which promotes the research and development of structure design of the composite leaf spring. It is believed that the developed algorithm is applicable to the structural design of a composite leaf spring with different structures, such as variable thickness and constant width.

## Figures and Tables

**Table 1:** Design requirements of the composite leaf spring.

Parameter	Value
Maximum mass, *M*_max_ (kg)	13
Bending stiffness, *k* (N·mm^−1^)	124 ± 12
Full load, *F*_1_ (N)	10,912
Limit load, *F*_2_ (N)	23,312
Safety coefficient, *N*	1.3
Span, *L* (mm)	1388
Maximum cross-sectional area, *S*_max_ (mm^2^)	2300
Maximum width, *b*_max_ (mm)	92
Maximum thickness, *h*_max_ (mm)	40
Allowable stress, [*σ*] (MPa)	673

**Table 2:** Design results of the composite leaf spring.

Parameter	Result
Cross-sectional area, *S* (mm^2^)	2000
Maximum thickness, *h*_max_ (mm)	37.1
Length of transition section, *l*_t_ (mm)	248
Maximum width, *b*_max_ (mm)	90
Maximum deflection, *w*_max_ (mm)	180
Bending stiffness, *k* (N·mm^−1^)	129.5
Maximum stress, *σ*_max_ (MPa)	580

**Table 3:** Technological parameters of the filament winding process.

Tension System (N/Layers)	Winding Speed (r·min^−1^)	Curing Process
60/4 + 40/4 + 20/3	3	85 °C/30 min + 95 °C/1 h + 105 °C/30 min + 120 °C/1 h

**Table 4:** Mechanical property parameters of E-glass fiber/epoxy composite material samples.

Parameter	Value
Elastic modulus along length direction, *E*_1_ (GPa)	52.5
Elastic modulus along thickness direction, *E*_2_ (GPa)	15
Elastic modulus along width direction, *E*_3_ (GPa)	15
Shear modulus along length-thickness direction, *G*_12_ (GPa)	5.0
Shear modulus along length-width direction, *G*_13_ (GPa)	5.0
Shear modulus along thickness-width direction, *G*_23_ (GPa)	1.8
Main Poisson’s ratio along length-thickness direction, *μ*_12_	0.3
Main Poisson’s ratio along length-width direction, *μ*_13_	0.3
Main Poisson’s ratio along thickness-width direction, *μ*_23_	0.1
Tensile strength along length direction, *X*_1_ (MPa)	1300
Compressive strength along length direction, *Y*_1_ (MPa)	875
Tensile strength along thickness direction, *X*_2_ (MPa)	48
Compressive strength along thickness direction, *Y*_2_ (MPa)	141
Tensile strength along width direction, *X*_3_ (MPa)	48
Compressive strength along width direction, *X*_3_ (MPa)	141
Interlaminar shear strength, *σ*_ILSS_ (MPa)	74.7
Longitudinal and transverse shear strength, *σ*_τ±45°_ (MPa)	48.7

**Table 5:** Comparison between the finite element (FE) results of the composite leaf spring under full and limit loading conditions and the material strength requirement.

Parameter	FE Result (MPa)		Material Strength Requirements (MPa)
	Under Full Loading Condition	Under Limit Loading Condition	
Tensile strength along length direction	577.9	605.2	1000
Compressive strength along length direction	579.2	602.4	673.1
Tensile strength along thickness direction	16.2	35.7	34.6
Compressive strength along thickness direction	16.1	31.1	108.5
Tensile strength along width direction	13.2	15.2	34.6
Compressive strength along width direction	13.6	16.6	108.5
Longitudinal and transverse shear strength, *σ*_τ±45°_	30.0	36.9	37.5
Interlaminar shear strength, *σ*_ILSS_	50.8	57.0	57.5

## Data Availability

The data presented in this study are available on request from the corresponding author.

## Appendix A. Measurement of the Mechanical Property Parameters of E-Glass Fiber/Epoxy Composite Material Samples

In order to obtain the mechanical property parameters as shown in Table 3, the E-glass fiber/epoxy composite material samples with 40% volume fraction of fiber were manufactured using the hot-pressing process. The prepreg with good quality was prepared by mixing the E-glass fiber and epoxy resin system into a glue solution, which was arranged by a wet winding machine. The prepreg was cut into the layer with a size of 250 × 300 mm, and 15 layers of prepreg were laid. The uncured sample was put into the mold and cured at the temperature level of 100 °C for 15 min, and 120 °C for 2 h, respectively. Subsequently, it was naturally cooled down to room temperature after pressurization. The composite samples with good forming quality were obtained. According to the requirement of different experimental setups, the samples with different shapes and sizes were prepared: (a) the overall dimensions of 250 × 25 × 3 mm, (b) the overall dimensions of 140 × 6 × 3 mm, (c) the cross-section dimensions of 250 × 25 mm, and the thickness of [+45/−45]_5S_ indicating five symmetrical plies, (d) the double V shape gap with the dimensions of 76 × 19 × 3 mm, (e) the cylinder shape with square section, and side length of 15 mm and height of 19 mm, and (f) the “T” shape with length of top surface of 35 mm, length of bottom surface of 20 mm, left side length and right side length of upper part of 7.5 and 8 mm respectively, and the thickness of 15 mm.

After the material preparation, the mechanical property parameters of E-glass fiber/epoxy composite material samples were measured [47,48,50]. Specifically, the elastic modulus, *E*_1_, along the length direction of the sample, Poisson’s ratio, *μ*_12_, and the tensile strength, *X*_1_, were measured via the transverse tensile test using a universal testing machine, where the sample a was adhered to the metal end and loaded along the length direction of the sample under the crosshead displacement control at a rate of 1.0 mm·min^−1^ until failure. The elastic modulus, *E*_2_, along the thickness direction of the sample and the tensile strength, *X*_2_, were measured via the interlaminar tensile test, where the sample e was loaded along the thickness direction under the displacement control at a rate of 0.1 mm·min^−1^, as the sample e with the cylinder shape required a lower loading rate than previous samples to ensure the sufficient loading time. The elastic modulus, *E*_3_, along the width direction of the sample, Poisson’s ratio, *μ*_13_, *μ*_23_, and the tensile strength, *X*_3_, were measured via the longitudinal tensile test, where the sample a was loaded along the width direction of the sample under the displacement control at a rate of 1.0 mm·min^−1^. The compressive strength *Y*_1_, *Y*_2_, and *Y*_3_ were measured via the compression performance test, where the sample b was loaded along the length, thickness, and width directions of the sample under the displacement control at a rate of 1.0 mm·min^−1^, respectively. The interlaminar shear modulus, *G*_12_, *G*_23_, were measured via the interlaminar shear test, where the sample d was loaded along the width and length directions under the displacement control at a rate of 1.0 mm·min^−1^. The shear modulus *G*_13_ were measured via the in-plane shear test, where the sample c was loaded along the thickness direction of the sample under the displacement control at a rate of 1.0 mm·min^−1^. The interlaminar shear strength, *σ*_ILSS_, was measured via _the_ interlaminar shear strength test, where the sample f was loaded along the length direction of the upper part under the displacement control at a rate of 5.0 mm·min^−1^, as the sample f with “T” shape required a higher loading rate than other samples to reach failure. The longitudinal and transverse shear strength, *σ*_τ±45°_, was measured via the longitudinal transverse shear test, where the sample c was loaded along a direction of 45 degrees to sample length under the displacement control at a rate of 1.0 mm·min^−1^.
